# *Saussurea involucrata CML6* Enhances Freezing Tolerance by Activating Antioxidant Defense and the CBF-COR Pathway in Plants

**DOI:** 10.3390/plants14152360

**Published:** 2025-08-01

**Authors:** Mengjuan Hou, Hui Kong, Jin Li, Wenwen Xia, Jianbo Zhu

**Affiliations:** College of Life Science, Shihezi University, Shihezi 832003, China; mjhoushzu@163.com (M.H.); huikshzu@163.com (H.K.); lijin@shzu.edu.cn (J.L.)

**Keywords:** *Saussurea involucrata*, *CML* proteins, cold stress, *CBF*, abiotic stress

## Abstract

Low-temperature stress severely limits plant growth and reduces agricultural productivity. Calmodulin-like (CML) proteins are crucial calcium sensors in plant cold responses. Transcriptome analysis of cold-stressed *Saussurea involucrata* identified seven differentially expressed *CML* genes. qRT-PCR confirmed *that SiCML6* was strongly induced at 4 °C and −2 °C. Bioinformatics analysis showed that *SiCML6* encodes a transmembrane protein containing an EF-hand domain. This protein carries a signal peptide and shows the closest phylogenetic relationship to *Helianthus annuus* CML3. Its promoter contains ABA, methyl jasmonate (MeJA), and cold-response elements. *Arabidopsis* plants overexpressing *SiCML6* showed significantly higher survival rates at −2 °C than wild-type plants. Under freezing stress, *SiCML6*-overexpressing lines exhibited reduced malondialdehyde content, relative electrolyte leakage, and ROS accumulation (H_2_O_2_ and O_2_^−^), along with increased proline, soluble sugars, soluble proteins, and total antioxidant capacity (T-AOC). *SiCML6* elevated the expression of cold-responsive genes *CBF3* and *COR15a* under normal conditions and further upregulated *CBF1/2/3* and *COR15a* at 4 °C. Thus, low temperatures induced *SiCML6* expression, which was potentially regulated by ABA/MeJA. *SiCML6* enhances freezing tolerance by mitigating oxidative damage through boosted T-AOC and osmoprotectant accumulation while activating the CBF-COR signaling pathway. This gene is a novel target for improving crop cold resistance.

## 1. Introduction

Low-temperature stress is one of the major abiotic stresses encountered by plants during growth and can lead to cell membrane damage, reactive oxygen species (ROS) bursts, and metabolic disorders. This significantly affects plant growth, development, yield, and quality [1]. Therefore, plants must sense and respond to low-temperature stress through complex regulatory mechanisms to improve their cold tolerance. Calcium ions (Ca^2+^), which act as ubiquitous second messengers, play crucial roles in plant growth, development, and responses to environmental stress. When plants perceive changes in their environment, the concentration of intracellular calcium ions undergoes rapid and spatially specific changes. These calcium ion signals can be detected by different calcium sensor proteins and decoded into downstream signals to trigger plant responses [2,3]. To date, four main categories of calcium sensor proteins have been identified: calcium-dependent protein kinases (CDPKs), calcineurin B-like (CBL) proteins, calmodulins (CaMs), and calmodulin-like (CML) proteins [4,5]. Among them, calmodulin is the most extensively studied calcium sensor protein and is found in all branches of eukaryotes. In contrast to the conserved and limited number of calmodulins, plants have evolved numerous calmodulin-like proteins to adapt to environmental changes. Calmodulin-like (CML) proteins contain a conserved calcium-binding domain (also known as the EF-hand), which is typically characterized by a helix–loop–helix structure [6]. Upon binding to calcium ions, these proteins undergo conformational changes that alter their enzymatic activity or regulate different targets through intermolecular interactions, thereby participating in the regulation of plant growth and development, as well as responses to biotic and abiotic stresses [4].

In plants, such as *Arabidopsis* and rice, the *CML* gene family is significantly larger than the conserved *CaM* family (for example, *Arabidopsis* contains 50 CML proteins versus seven CaMs) [7], suggesting that their functional diversification contributes to environmental adaptation. In recent years, considerable progress has been made in studying the roles of calmodulin-like (CML) proteins in plant resistance to abiotic stress. Research has shown that different *CML* genes have distinct functions in response to stress. For example, *AtCML9* overexpression reduces drought and salt tolerance in *Arabidopsis*, whereas the *cml9* mutant affects the expression of multiple stress-responsive genes and exhibits enhanced drought and salt tolerance and increased sensitivity to ABA [8]. The ShCML44 gene, isolated from a cold-tolerant wild tomato, significantly improves tomato tolerance to cold, drought, and salt stresses by enhancing antioxidant activity, water retention, and regulating stress-related gene expression [9]. CML proteins have also been found to be involved in stress responses in leguminous and cucurbitaceous species. In alfalfa, *MtCML40* negatively regulates salt tolerance in *Medicago truncatula* by inhibiting MtHKT-dependent Na^+^ efflux [10], and in melon, calmodulin-like protein *CmCML13* is a multifunctional protein that significantly enhances salt and drought tolerance in transgenic *Arabidopsis* by reducing shoot sodium content and localizing to multiple subcellular compartments [11]. In monocot crops, OsCML16 is a direct target of OsERF48, where ERF (ethylene-responsive factor) refers to a family of transcription factors that play crucial roles in plant responses to various environmental stresses and developmental processes. Their interaction promotes root growth and enhances drought tolerance in rice [12]. In wheat, *TaCML20* significantly increases tiller number and grain weight by regulating the accumulation of water-soluble carbohydrates and the expression of related genes, playing an important role in drought stress [13]. Different *CML* genes within the same species can also have opposing functions; for example, both *AtCML37* and *AtCML42* in *Arabidopsis* are involved in the drought stress response, but their roles are nearly opposite [14]. Notably, some *CML* genes (such as *AtCML42*), as multifunctional Ca^2+^ sensors, can link Ca^2+^ signaling with other pathways (such as jasmonic acid, JA), thus playing roles in both biotic (e.g., insect defense) and abiotic stress responses [15]. These studies indicate that *CML* genes play complex and diverse roles in plant responses to abiotic stress.

*Saussurea involucrata* is a rare medicinal plant that grows in high-altitude, cold regions and possesses excellent characteristics, such as cold and drought resistance. Its unique growing environment has enabled it to develop a highly efficient low-temperature adaptation mechanism over the course of long-term evolution [16], resulting in the evolution of many unique stress-resistant functional genes [16,17,18]. This aligns with patterns observed in extremophytes like *Sporobolus stapfianus*, where lineage-specific gene expansions underpin dehydration tolerance [19]. However, there are currently few reports on the calmodulin genes in *S. involucrata*. In this study, *CML6* from *S. involucrata* was used as the research subject. Through systematic analysis of the expression patterns of *SiCML6* under cold stress, its protein structural features, and its regulatory network, coupled with overexpression of this gene in the model plant *A. thaliana* and combined physiological, biochemical, and molecular biological analyses, the aim was to explore its function under cold stress and reveal its mechanism of plant cold tolerance. This study not only provides new evidence for a deeper understanding of the role of plant calmodulins in response to cold stress but also offers important theoretical support and genetic resources for breeding cold-resistant plant varieties and for conserving and rationally utilizing *S. involucrata* resources by identifying this key cold-responsive gene.

## 2. Results

### 2.1. Expression Analysis of the CML Gene from Saussurea involucrata Under Low Temperature Stress

**Figure 1:**
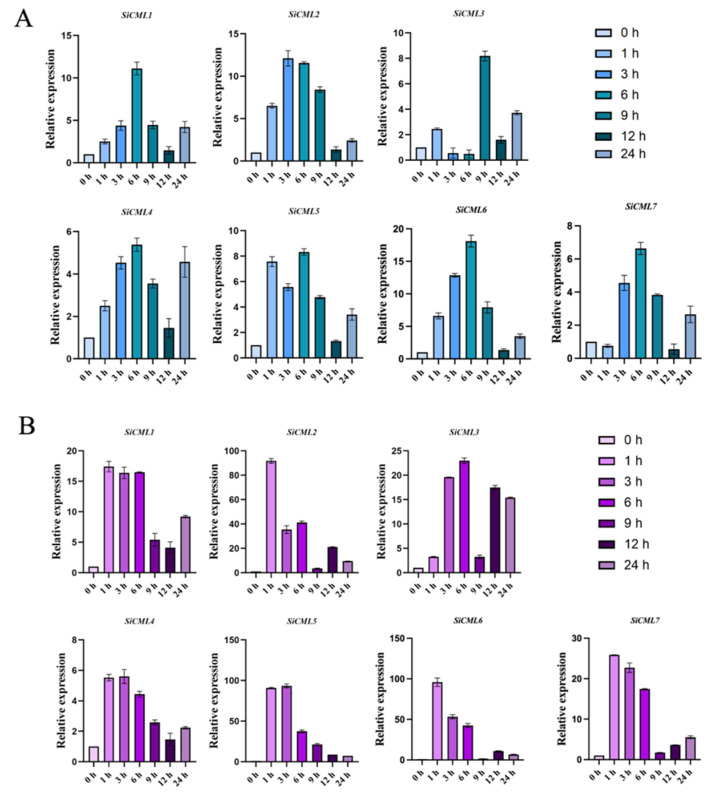
Temporal expression profiles of *S. involucrata CML* proteins under low-temperature stress. (**A**) Relative expression dynamics of *SiCML* proteins after treatment at 4 °C. (**B**) Relative expression dynamics of *SiCML* proteins after treatment at −2 °C. Data are presented as the mean ± standard deviation (mean ± SD, *n* = 3) from three biological replicates.

We validated the expression patterns of seven highly expressed *CML* genes in *S. involucrata* under cold stress using qRT-PCR. The results showed that all seven *CML* genes in *S. involucrata* were affected by cold stress (Figure 1). Under 4 °C stress, the expression levels of *SiCML1/4/5/6/7* peaked at 6 h. *SiCML6* was more sensitive to 4 °C stress in terms of expression level than the other six genes (Figure 1A). Under −2 °C stress, except for *SiCML3*, the expression levels of the other six *CML* genes in *S. involucrata* showed a trend of first increasing and then decreasing, among which *SiCML5* and *SiCML6* were more sensitive to −2 °C stress (Figure 1B). The expression level of *SiCML6* increased dramatically under both 4 °C and −2 °C stress, indicating that it may serve as a key regulatory factor involved in the cold response pathway of *S. involucrata*. Therefore, it was selected as a target gene for subsequent cold-resistance functional validation.

### 2.2. Bioinformatics Analysis of SiCML6

**Figure 2:**
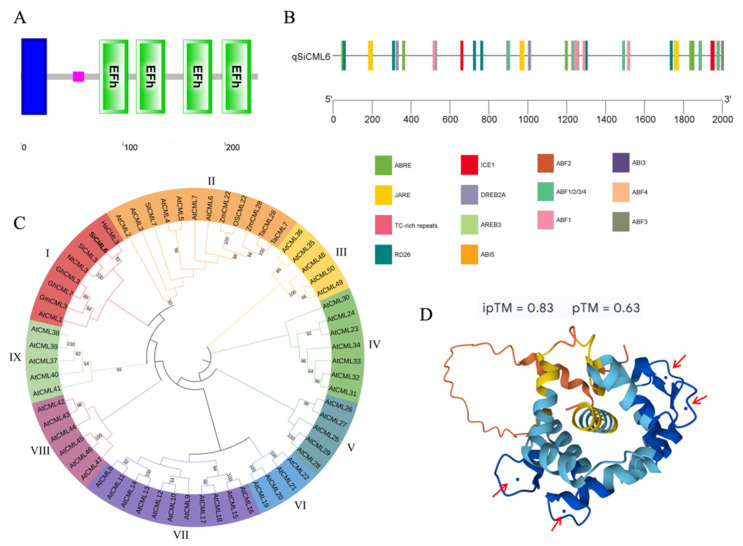
Analysis of SiCML6 protein domains, promoter cis-acting elements, phylogenetic tree, and calcium-binding affinity. (**A**) Conserved domain analysis of the SiCML6 protein (SMART). The blue box indicates the PEF superfamily (Penta-EF-hand) region, and the green boxes indicate the positions of the four EF-hand domains (aa 77–105, 113–141, 159–187, and 197–225). (**B**) Distribution of cis-acting elements in the *SiCML6* promoter. The key response elements include ABA response elements (ABREs, 5), methyl jasmonate response elements (JAREs, 6), ABA-related transcription factors (ABF1/2/3/4, ABI3/5, and AREB3), and cold response factor binding sites (ICE1, RD26, and DREB2A), with the numbers indicated in parentheses. (**C**) Phylogenetic tree of SiCML6 (maximum likelihood method, 1000 bootstrap replicates). SiCML6 is indicated in bold. Species abbreviations: At (Arabidopsis thaliana), Gh (cotton), Sl (tomato), Os (rice), Zm (maize), Nt (tobacco), and Ta (wheat). (**D**) Predicted calcium-binding capacity of SiCML6 (AlphaFold3). The red arrows indicate the calcium-binding sites.

The open reading frame of *SiCML6* was 699 bp in length, encoding a protein composed of 232 amino acids with a theoretical molecular weight of 26.28 kDa and an isoelectric point of 4.99. SMART conserved domain analysis revealed that the protein encoded by SiCML6 belongs to the Penta-EF-hand (PEF) superfamily and contains an EF-hand domain. This protein contains four calcium-binding sites located at amino acids 77–105, 113–141, 159–187, and 197–225 (Figure 2A). Computational analysis using a third-generation protein folding algorithm (AlphaFold3) indicated that SiCML6 has a strong calcium-binding capacity. Further calculations showed that the protein’s ability to bind two calcium ions was higher than its ability to bind a single calcium ion (Figure S2), and binding four calcium ions exhibited the highest affinity (Figure 2D). Additionally, SignalP-4.1 analysis of the SiCML6 protein revealed that there is a signal peptide at the N-terminus, and TMHMM analysis of its transmembrane domains showed that the protein contains one transmembrane domain (Figure S1). Promoter analysis (Figure 2B) indicated that the *SiCML6* promoter contains five ABA-responsive elements (ABREs) and six methyl jasmonate response elements (JAREs). Moreover, multiple ABA-related transcription factor binding sites were predicted, including ABF1 (14 sites), ABF2 (8), ABF3 (11), ABF4 (12), ABI3 (4), ABI5 (5), and AREB3 (5). Simultaneously, six binding sites for the cold acclimation-related transcription factor ICE1 were predicted, as well as two reported cold stress-related transcription factor binding sites for RD26 (8) and DREB2A (5). The presence of multiple stress-responsive elements suggests *SiCML6* may be integrated into complex stress response networks. Phylogenetic analysis (Figure 2C) revealed that the SiCML6 protein and *Arabidopsis* AtCML1 are on the same evolutionary branch. A comparison of CML proteins from other species revealed that SiCML6 is most closely related to CML3 from *Helianthus annuus*, which is consistent with the taxonomic classification of *S. involucrata* and *H. annuus*, both of which belong to the Asteraceae family. In addition, SiCML6 is closely related to CML3 in dicotyledonous plants, such as tobacco and tomato.

### 2.3. SiCML6 Increases the Survival Rate of Arabidopsis Under Low-Temperature Stress

**Figure 3:**
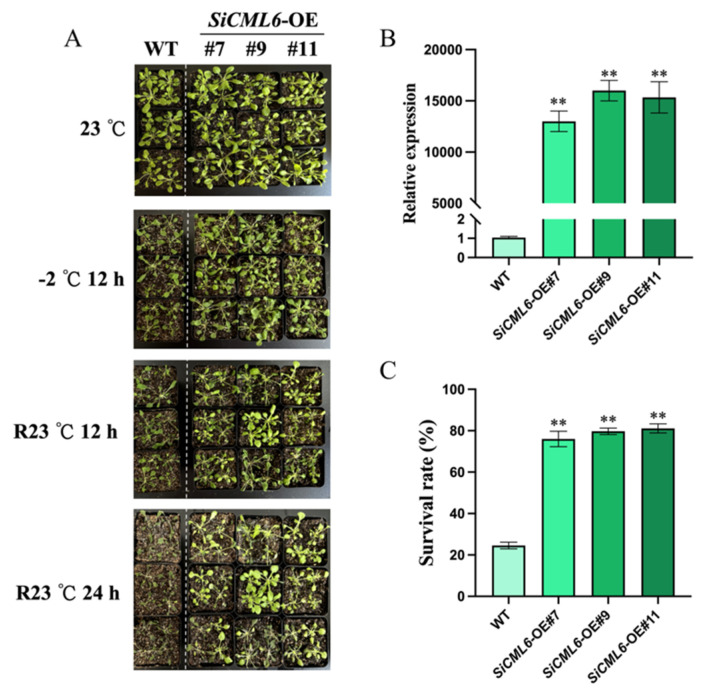
Enhanced cold tolerance in *SiCML6*-overexpressing *Arabidopsis* transgenic lines. (**A**) Phenotypes of wild-type (WT) and *SiCML6*-overexpressing (*SiCML6*-OE: #7, #9, and #11) Arabidopsis transgenic lines before cold treatment, after cold treatment, and during the recovery period. (**B**) qRT-PCR analysis of gene expression in WT and *SiCML6*-OE lines. (**C**) Survival rates of WT and *SiCML6*-OE lines after cold treatment. ** Denotes a significant difference at *p* < 0.01.

*SiCML6* is rapidly induced and expressed under stress at 4 and −2 °C. To verify the role of *SiCML6* in plant resistance to low-temperature stress, this study established heterologous overexpression lines of *SiCML6* in *Arabidopsis* for subsequent cold tolerance analysis (Figure 3A). First, three *SiCML6* overexpression lines (*SiCML6*-OE#7, *SiCML6*-OE#9, and *SiCML6*-OE#11) were selected by PCR (Figure S3) and qRT-PCR (Figure 3B). Low-temperature stress experiments were conducted on *Arabidopsis* plants two weeks after transplantation into potting soil. As shown in Figure 3A, under normal growth conditions, no significant differences in growth were observed between wild-type (WT) and *SiCML6*-OE plants. However, after 12 h of −2 °C cold stress, all *Arabidopsis* leaves exhibited wilting, with the degree of wilting in *SiCML6*-OE plants being less severe than that in WT plants. After all plants were returned to 23 °C for 12 h, some *SiCML6*-OE plants returned to normal growth, whereas the WT plants did not recover. After 24 h of recovery at 23 °C, almost all *SiCML6*-OE plants returned to normal growth, whereas nearly all WT plants died. Statistical analysis showed that the survival rate of *SiCML6*-OE plants after low-temperature treatment was significantly higher than that of WT plants (Figure 3C). These results indicate that *SiCML6* overexpression enhances the survival rate of *Arabidopsis* under freezing stress.

### 2.4. Analysis of Physiological Indices Related to Cold Tolerance Assessment in SiCML6 Transgenic Arabidopsis

**Figure 4:**
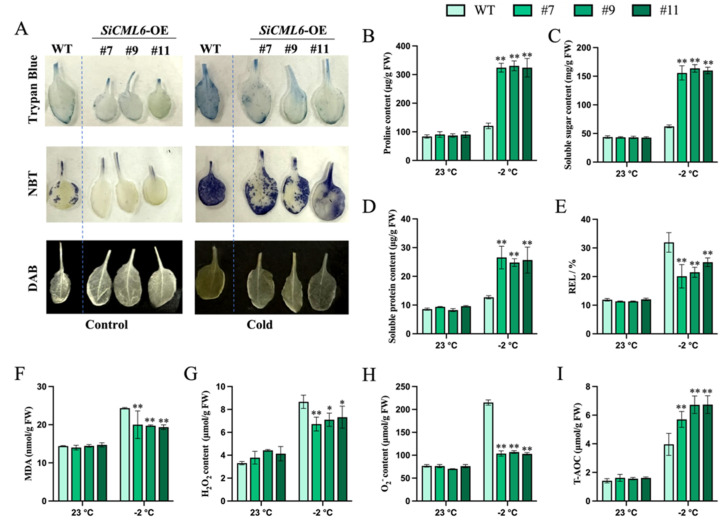
Physiological responses of WT and *SiCML6*-OE lines to freezing stress. (**A**) Phenotypes of WT and *SiCML6*-OE transgenic lines stained with trypan blue, DAB, and NBT before and after −2 °C stress. (B–F) Pro content (**B**), SS content (**C**), SP content (**D**), REL (**E**), MDA content (**F**), H_2_O_2_ content (**G**), O_2_^−^ content (**H**), and T-AOC activity (**I**) of WT and *SiCML6*-OE lines before and after −2 °C stress. * Indicates a significant difference at *p* < 0.05; ** indicates a highly significant difference at *p* < 0.01.

To elucidate the physiological basis for the enhanced survival of *SiCML6*-OE plants under −2 °C stress, we first assessed cellular viability and then systematically analyzed key markers of stress adaptation: osmotic adjustment, membrane integrity, oxidative stress, and antioxidant capacity. Trypan blue staining revealed that 12 h at −2 °C resulted in larger stained areas on WT leaves than on OE lines (Figure 4A), indicating markedly fewer dead cells in the *SiCML6*-OE plants after freezing. To assess osmotic adjustment—a key mechanism for maintaining cellular hydration under freezing stress—we quantified proline (Pro), soluble sugars (SSs), and soluble proteins (SPs). While no genotypic differences were observed under normal conditions, *SiCML6*-OE lines accumulated significantly higher levels of all three osmoprotectants compared to WT following −2 °C treatment (Figure 4B–D). This accumulation is crucial for stabilizing cellular structures and mitigating dehydration damage during freezing. Given that membrane damage is a primary consequence of freezing injury, we measured relative electrolyte leakage (REL, an indicator of membrane permeability) and malondialdehyde (MDA, a marker of lipid peroxidation). Both parameters were comparable between genotypes under control conditions. However, after −2 °C stress, *SiCML6*-OE plants exhibited significantly lower REL and MDA levels than WT (Figure 4E,F), indicating superior membrane integrity in the transgenic lines under freezing stress. Excessive ROS accumulation is a hallmark of oxidative stress triggered by cold, leading to cellular damage. We therefore monitored the in situ accumulation of superoxide anion (O_2_^−^) and hydrogen peroxide (H_2_O_2_) using nitro blue tetrazolium (NBT) and 3,3′-diaminobenzidine (DAB) staining, respectively. After −2 °C treatment, detached leaves from *SiCML6*-OE plants showed markedly fainter NBT and DAB staining compared to WT (Figure 4A). Quantitative assays corroborated these findings, revealing significantly lower levels of both H_2_O_2_ and O_2_^−^ in the transgenic lines post-stress (Figure 4G,H). This reduction in ROS suggests enhanced control over oxidative burst in *SiCML6*-OE plants. To understand the mechanism underlying reduced ROS levels, we evaluated the total antioxidant capacity (T-AOC), which reflects the integrated ability to scavenge ROS. While T-AOC was similar between genotypes under control conditions, it was significantly higher in *SiCML6*-OE lines than in WT following −2 °C stress (Figure 4I). This boost in antioxidant capacity provides a direct explanation for the observed attenuation of ROS accumulation. Collectively, these physiological analyses demonstrate that *SiCML6* enhances freezing tolerance through coordinated mechanisms: (1) promoting the accumulation of osmoprotectants (Pro, SSs, and SPs) to maintain cellular hydration and stability; (2) boosting total antioxidant capacity (T-AOC) to mitigate oxidative damage by suppressing ROS accumulation (H_2_O_2_, O_2_^−^). This dual action synergistically preserves membrane integrity (reduced REL and MDA) and cellular homeostasis under freezing stress.

### 2.5. SiCML6 Regulates Downstream Genes of the Arabidopsis Low-Temperature Response Pathway

To investigate how *SiCML6* overexpression influences key components of the low-temperature signaling pathway, we monitored the transcript levels of CBF (C-repeat binding factor), family genes (*AtCBF1*, *AtCBF2*, and *AtCBF*3), and the cold-responsive gene *AtCOR15a* by qRT-PCR before and after exposure to 4 °C. Under normal growth conditions (23 °C), the relative expression levels of *AtCBF1* and *AtCBF2* did not differ between WT and *SiCML6*-OE plants. However, *AtCBF3* expression was markedly higher in *SiCML6*-OE lines than in WT (*p* < 0.01), and *AtCOR15a* was also significantly upregulated (*p* < 0.05). Following 3 h of cold stress at 4 °C, the transcript abundances of *AtCBF1*, *AtCBF2,* and *AtCBF3* were all significantly elevated in *SiCML*6-OE plants compared with WT (*p* < 0.05). After 24 h at 4 °C, *AtCOR15a* expression was extremely higher in *SiCML6*-OE lines than in WT (*p* < 0.01). These results suggest that *SiCML6* enhances *Arabidopsis* freezing tolerance by potentiating the transcriptional activity of the CBF-COR signaling pathway, particularly through upregulating *AtCBF3* and *AtCOR15a*.

## 3. Discussion

Freezing severely restricts both the germination of crop seeds and their range of suitable cultivation, making the functional validation and characterization of antifreeze genes highly significant. In this study, we analyzed the function of *SiCML6* in plant responses to low-temperature stress. The results show that *SiCML6* is significantly upregulated under 4 °C and −2 °C stress treatments, possesses EF-hand domains and Ca^2+^ binding sites, and its promoter is rich in stress-responsive elements. Transgenic validation indicated that *SiCML6* significantly enhanced the freezing tolerance of Arabidopsis by accumulating osmoprotectants, boosting antioxidant defense, and synergizing with the CBF-COR signaling pathway.

The expression patterns of genes under stress are often closely related to their function during such conditions [20]. In this study, seven *CML* proteins were identified from the cold-stress transcriptome of *Saussurea involucrata*. The presence of multiple cold-responsive CML proteins in this alpine plant (Figure 1) implies functional specialization rather than simple redundancy, which aligns with reports that different CML proteins can exert distinct or even opposing roles during stress responses [2]. Subsequent qRT-PCR analyses revealed that *SiCML6* is markedly upregulated under both 4 °C and –2 °C treatments, indicating that *SiCML6* likely plays an important role in low-temperature responses. Bioinformatics analysis showed that SiCML6 features a typical PEF superfamily EF-hand domain and four Ca^2+^ binding sites, which are characteristics of a calcium sensor [21]. Its N-terminal signal peptide and transmembrane domain (amino acids 2–24) suggest possible subcellular localization or membrane-associated signal transduction functions, possibly participating in transmembrane calcium signaling triggered by cold [22]. Phylogenetic analysis revealed high homology with sunflower CML3, consistent with the classification of Asteraceae plants and providing evidence of a conserved gene function. Additionally, SiCML6 protein is closely related to maize CML22 and CML28, of which ZmCML28 is significantly induced under abiotic stress [23], implying that *SiCML6* may also play a significant role in abiotic stress responses. Promoter analysis revealed that *SiCML6* expression is induced by the plant hormones ABA and methyl jasmonate and is associated with ABA-related and cold-induced transcription factors. The presence of ABA and MeJA response elements in the *SiCML6* promoter (Figure 2B) suggests potential hormonal regulation, consistent with reports that some CML proteins integrate calcium signaling with hormone pathways [15]. The promoter analysis indicates *SiCML6* may be regulated by multiple stress signaling pathways, positioning it as a node connecting calcium signaling with hormonal and cold response networks. Calcium-binding proteins undergo conformational changes upon binding to calcium ions, relieving autoinhibition and enabling biological function [24]. In this study, SiCML6 exhibited a stronger ability to bind four calcium ions than two or one, which supports this classical hypothesis. This also indicates that after binding one calcium ion, SiCML6’s affinity for binding more is enhanced. Therefore, we speculate that *SiCML6* is an important gene involved in the low-temperature response in plants.

To further verify whether *SiCML6* participates in freezing resistance, we performed freezing tolerance assays on *Arabidopsis* plants that overexpressed *SiCML6*. The transgenic lines had significantly higher survival rates under −2 °C low-temperature stress compared to wild-type plants, demonstrating that overexpression of *SiCML6* enhances *Arabidopsis* tolerance at −2 °C. Measurements of physiological and biochemical indicators showed that after cold stress, the Pro content in the overexpression lines was significantly higher than that in the WT, a finding consistent with previous studies in which Pro and SSs significantly accumulated in *Arabidopsis* overexpressing *VaCIPK18* under low temperature (helping maintain membrane stability and osmotic balance) [25] and with established roles of osmoprotectants in membrane stabilization across plants [26], while notably, proline also acts as a molecular chaperone to protect enzyme structure under stress [27]. The increases in MDA content and REL were lower in the transgenic lines than in the WT, indicating less membrane damage under cold stress in *SiCML6*-OE plants than in WT. Numerous studies have shown that cold stress induces the production of ROS, and excessive ROS production results in oxidative damage to organelles, proteins, DNA, and membrane lipids. In this study, the *SiCML6*-OE lines exhibited lower ROS levels and more pronounced increases in T-AOC activity under cold stress, suggesting that *SiCML6* enhances the antioxidant system to scavenge ROS, thereby reducing ROS-related oxidative damage and improving cold tolerance in Arabidopsis. This finding is in strong agreement with those of other studies. For example, after cold treatment, tomato fruits overexpressing *SlCML37* showed lower H_2_O_2_ levels, and MDA content and electrolyte leakage were significantly lower than those in the WT, indicating that overexpression of *SlCML37* increases fruit cold tolerance [28]. In mangrove species, *MpCML40* enhances Arabidopsis tolerance to salt stress by alleviating oxidative damage [29]. In wild soybean, *GsCML27* is upregulated under bicarbonate, salt, and osmotic stresses [30]. Overexpression of *OsCML4* in rice also increases resistance to abiotic stress [31]. In Arabidopsis, the cml20 mutant accumulates more ROS and shows downregulated antioxidant gene APX2 expression under drought and ABA treatment [32]. These studies demonstrate that CML proteins help mitigate oxidative damage by coordinating antioxidant defense, a conserved trans-species strategy. The changes in these physiological indicators were consistent with the enhanced low-temperature tolerance exhibited by the overexpression lines, further confirming the important role of *SiCML6* in improving plant cold resistance. Although this study focuses on cold tolerance, multiple CML protein family members are known to confer cross-stress tolerance. For example, tomato *ShCML44* enhances resistance to cold, drought, and salt stresses [9], and the melon *CmCML13* gene improves salt and drought tolerance in *Arabidopsis* [11]. These findings suggest that *SiCML6* may possess similar broad-spectrum functions and warrant further investigation.

Furthermore, this study found that *Arabidopsis* overexpressing SiCML6 activated the expression of downstream cold-inducible genes *AtCBF1*, *AtCBF2*, *AtCBF3*, and *AtCOR15a*. The *CBF* gene family regulates cold response through a well-characterized mechanism. CBF proteins bind to *COR* gene promoters, activating their expression and consequently enhancing cold tolerance [33]. Previous studies have shown that the overexpression of grape *CML21*v2/v4 splice variants enhances the transcriptional responses of cold-induced genes, such as *AtCOR47*, resulting in significantly improved freezing survival rates [34]. Although tomato *SlCML39* mainly responds to heat stress, its overexpression also affects the expression of heat-responsive genes such as *KIN1* and *RD29B* [35]. *SiCML6* likely acts as a calcium decoder that bridges cold sensing to CBF activation, a functional role analogous to the CBL1-CIPK7 signaling pathway in mediating cold responses [36], thereby highlighting a conserved paradigm wherein calcium sensors enhance the activity of CBF regulons [3]. Although the direct target of SiCML6 remains to be identified, its calcium-sensing capability and ability to activate CBF expression suggest it may function early in cold signal transduction, potentially through calcium-mediated activation of CBF transcription factors or their regulators. These studies show that CML proteins may participate in different stress responses through interactions with the CBF-COR pathway. Based on our findings, *SiCML6* may enhance plant cold tolerance by regulating the CBF-COR pathway and activating cold response genes, providing important clues for exploring the molecular regulatory mechanisms.

**Figure 5:**
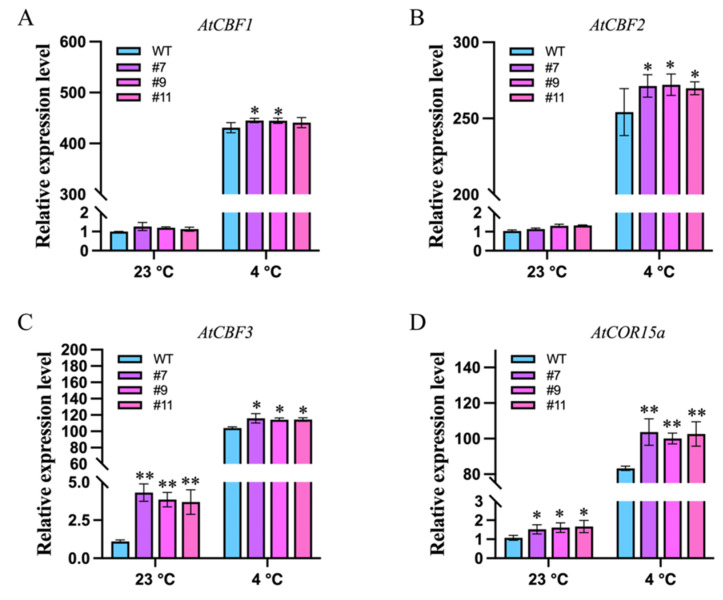
Effects of *SiCML6* overexpression on the expression of low-temperature-responsive pathway genes in *Arabidopsis*. Relative transcript levels of CBF family genes *AtCBF1* (**A**), *AtCBF2* (**B**), *AtCBF3* (**C**), and the cold-responsive gene *AtCOR15a* (**D**) in WT and *SiCML6*-OE lines under normal temperature (23 °C) and 4 °C cold stress were analyzed by qRT-PCR. Data are presented as mean ± SE (*n* = 3). Asterisks indicate significant differences: * *p* < 0.05; ** *p* < 0.01.

In summary, this study revealed the function of *SiCML6* from *S. involucrata* in plant cold tolerance. Through mechanisms including enhanced antioxidant enzyme activity, reduced ROS levels, maintained membrane stability, and activation of downstream cold-inducible genes in the CBF-COR pathway, *SiCML6* synergistically enhances plant freezing resistance. These results not only enrich research on the role of plant calmodulin-like proteins in low-temperature stress responses but also provide a promising genetic resource for utilizing genetic engineering to cultivate cold-resistant plant varieties, which has important theoretical and practical implications. *SiCML6* may enhance CBF-COR pathway activity through multiple mechanisms: (1) as a calcium sensor, it could directly modulate CBF regulators in response to cold-induced calcium signals, and/or (2) by maintaining redox homeostasis (Figure 4), it may create cellular conditions favorable for cold signal transduction. The early upregulation of CBF3 in unstressed transgenic plants (Figure 5C) suggests *SiCML6* may prime the cold response machinery. However, the precise molecular regulatory mechanisms of *SiCML6* in plant cold tolerance (such as its interaction with the CBF-COR pathway) and its relationship with other cold-resistance-related genes remain to be explored. Such studies will facilitate a more comprehensive understanding of plant cold tolerance mechanisms and offer more robust theoretical support for the genetic improvement of plant cold resistance.

## 4. Materials and Methods

### 4.1. Plant Materials and Cultivation Conditions

Tissue-cultured seedlings of *Saussurea involucrata* Kar. et Kir. used in the experiment were provided and preserved in our laboratory. *Arabidopsis thaliana* seeds were sterilized and sown on 1/2 Murashige and Skoog (MS) solid medium and placed in an incubator at 23 °C under light for 7–9 days to allow for germination and growth. Once the seedlings reached the appropriate stage, they were transplanted into plastic pots filled with a mixture of nutrient soil and vermiculite at a 3:1 (*v*/*v*) ratio. *Arabidopsis* plants were cultivated in controlled-environment growth chambers or light incubators, with the temperature set at 23 °C. The light cycle was maintained at 12 h light/12 h dark during vegetative growth and 14 h light/10 h dark during reproductive growth.

### 4.2. Expression Profile Analysis of S. involucrata Calmodulin-like Protein Genes Under Low-Temperature Stress

Based on previously published transcriptome data of *S. involucrata* leaves under 4 °C and −2 °C low-temperature stress from our laboratory [37], seven calmodulin-like protein genes were identified using PF0036 (BCV = 0.2, logFC ≥ 2, and FDR < 0.001), which were named *SiCML1*–*SiCML7*. Gene-specific qRT-PCR primers were designed (sequences are provided in the Supplementary Materials, Table S1). Tissue-cultured *S. involucrata* seedlings were treated at 4 °C and −2 °C in light incubators. Samples collected at 0, 1, 3, 6, 9, 12, and 24 h were immediately flash-frozen in liquid nitrogen and stored at −80 °C. Total RNA was extracted from *S. involucrata* samples across the treatment groups and time points, reverse-transcribed into cDNA, and stored at −20 °C. qRT-PCR was performed using SYBR Green I Master Mix (following the manufacturer’s protocol), with *GAPDH* as the internal reference. Relative expression levels were calculated using the 2^−ΔΔCt^ method [38].

### 4.3. Cloning and Sequence Analysis of SiCML6

Using cDNA from *S. involucrata* as a template, the full-length CDS of *SiCML6* was amplified using primers *SiCML6*-F and *SiCML6*-R (primer sequences are shown in Table S1). The amplified product was inserted into the pMD19-T vector (TaKaRa, Dalian, China) for sequencing. The correctly sequenced *SiCML6* fragment was inserted into the pCAMBIA2300 vector by restriction ligation and transformed into Agrobacterium GV3101 via electroporation. Conserved domain analysis was performed using the SMART website (http://smart.embl-heidelberg.de/ assessed on 7 June 2025). SignalP (https://services.healthtech.dtu.dk/service.php?SignalP-5.0 accessed on 7 June 2025) was used to analyze the protein’s signal peptide [39], and TMHMM (https://services.healthtech.dtu.dk/service.php?DeepTMHMM accessed on 7 June 2025) was used to predict the transmembrane domains. The PlantCARE online tool [40] (http://bioinformatics.psb.ugent.be/webtools/plantcare/html/ accessed on 2 May 2025) was used to analyze cis-acting elements in the *SiCML6* promoter region. The ability of *SiCML6* to bind calcium ions was predicted using the third-generation protein folding algorithm AlphaFold3 (https://alphafold.ebi.ac.uk/ accessed on 6 May 2025). The predicted structure (PDB format) was visualized and binding sites analyzed in PyMOL (v2.5.2). Amino acid sequences of 50 CML proteins were downloaded from the *Arabidopsis* database, and CML protein sequences from the following species were obtained from the Phytozome database: sunflower (*Helianthus annuus*) HaCML3 (XP_021996713.1); soybean (*Glycine max*) GmCML3 (XP_003549743.1); tomato (*Solanum lycopersicum*) SlCML3 (XP_004242760.2) and SlCML7 (XP_069147216.1); rice (*Oryza sativa Japonica Group*) OSCML22 (NP_001406530.1); wheat (*Triticum aestivum*) TaCML7 (XP_044361313.1) and TaCML28 (XP_044443608.1); tobacco (*Nicotiana tabacum*) NtCML3 (XP_016440228.1); cotton (*Gossypium hirsutum*) GhCML3 (XP_016720830.1) and GhCML7 (XP_040954926.1); and maize (*Zea mays*) ZmCML28 (PWZ46430.1) and ZmCML22 (PWZ41168.1). These sequences, together with the SiCML6 amino acid sequence, were subjected to multiple sequence alignment using TBtools software (v2.222) [41]. The maximum-likelihood (ML) tree was constructed using IQ-TREE (v2.2.0) with the JTT + G4 model (best-fit model selected by ModelFinder) and 1000 ultrafast bootstrap replicates. The tree was visualized and annotated in iTOL (v6).

### 4.4. Transformation of Arabidopsis Thaliana

According to the method described by Zhang et al. (2006) [42], the *SiCML6* gene was transformed into *Arabidopsis thaliana* using the floral dip method to obtain T0 generation plants. The harvested T0 seeds were screened for 100 mg/L kanamycin resistance and subjected to PCR to confirm transgenic positives. Ultimately, homozygous T3 generation *SiCML6*-overexpressing lines were obtained through continuous propagation and used for low-temperature stress experiments.

### 4.5. Analysis of Cold Resistance in Transgenic Plants

T3-generation *SiCML6*-OE plants and WT with uniform growth status after 4 weeks were cultivated in a growth chamber (23 °C, 14 h light/10 h dark) until the early bolting stage. To minimize stress from sudden temperature changes, all plants were pre-adapted to a 4 °C environment (12 h light/12 h dark) for 24 h. Subsequently, each group of plants was transferred to a −2 °C low-temperature illuminated incubator (relative humidity ≥ 60%, light intensity 120 μmol·m^−2^·s^−1^) for continuous 12 h cold stress treatment. After treatment, the plants were sequentially placed in a 4 °C environment for 12 h and then returned to normal cultivation at 23 °C for recovery. Survival rates were determined based on new-leaf emergence and plant uprightness.

### 4.6. Histochemical Staining

To evaluate the effects of SiCML6 on cell death and reactive oxygen species (O_2_^−^, H_2_O_2_) accumulation in Arabidopsis thaliana leaves, trypan blue, nitroblue tetrazolium (NBT), and 3,3′-diaminobenzidine (DAB) were used to stain the leaves of wild-type (WT) and SiCML6-overexpressing lines before and after low-temperature stress. The specific experimental procedures followed the instructions provided with the kits purchased from Beijing Leigen Biotechnology (product codes: Trypan blue, DP0180; NBT, DP0045; DAB, DP0040).

### 4.7. Determination of Physiological and Biochemical Indicators

All assays were performed on the 4th-6th rosette leaves from 4-week-old plants. Samples were collected before (0 h) and immediately after 12 h at −2 °C stress. The relative electrolyte leakage (REL) was measured using a conductivity meter (EC 215, Markson Science, USA) following the protocol described by Xia et al. (2021) [43]. The leaf samples were immersed in deionized water, and the electrical conductivity was recorded before and after boiling the samples. The quantification of osmotic adjustment substances was performed based on the methods described by Kong et al. (2011) and Wang et al. (2022) [44,45]. Leaves were collected before and after the−2 °C stress treatment. Soluble sugars were measured using the anthrone–sulfuric acid method (620 nm), and soluble proteins were determined using the Bradford method (595 nm) [46]. Proline content was assessed using the acidic ninhydrin method (520 nm). Leaf tissue (0.1 g) was homogenized in 3% sulfosalicylic acid. After centrifugation, the supernatant reacted with acid–ninhydrin and glacial acetic acid (1:1:1 *v*/*v*) at 100 °C for 1 h. Absorbance of the toluene-extracted chromophore was read at 520 nm. Proline concentration was calculated using a standard curve (0–100 μg/mL). Oxidative stress indicators, including malondialdehyde (MDA), superoxide anion (O_2_^−^), hydrogen peroxide (H_2_O_2_) content, and total antioxidant capacity (T-AOC), were measured using Solarbio assay kits (catalogue numbers: MDA, BC0025; O_2_^−^, BC3590; H_2_O_2_, BC3595; T-AOC, BC1315), according to the manufacturer’s instructions.

### 4.8. Statistical Analysis

All data were based on the experimental results of three independent biological replicates and are presented as mean ± standard error (mean ± SE). One-way ANOVA was performed using SPSS 22.0 software, and the significance of intergroup differences was verified using Tukey’s post hoc test. Statistical significance was defined as *p* < 0.05 (*) and *p* < 0.01 (**). Graphs were generated using GraphPad Prism version 9.0.

## 5. Conclusions

In summary, *SiCML6* was induced at 4 °C and −2 °C and played a crucial role in plant resistance to low temperatures. *SiCML6* increased the content of osmotic protectants and the total activity of antioxidant enzymes under freezing conditions, thereby reducing the levels of ROS and the degree of membrane damage. *SiCML6* significantly enhanced the expression of *CBF3* and *COR15a* under normal conditions and cold acclimation-related genes under freezing conditions. Ultimately, *SiCML6* improved the freezing tolerance of *Arabidopsis*. *SiCML6* is an important genetic resource for enhancing plant freezing resistance.

## Data Availability

The data supporting the findings of this study are available in the Supplementary Materials section and from the corresponding author upon reasonable request.

## Supplementary Materials

The following supporting information can be downloaded at https://www.mdpi.com/article/10.3390/plants14152360/s1: Table S1: Primers used in this study; Figure S1: Signal peptide (A) and transmembrane domain analysis (B) of SiCML6; Figure S2: Molecular docking of SiCML6 with varying numbers of calcium ions; Figure S3: Characterization of transgenic *Arabidopsis thaliana* through DNA PCR; numbers 1–22 indicate individual transgenic plant lines; Sequence S1: *SiCML6* CDS sequence; and Sequence S2: SiCML6 Protein sequence.
