# Supplementary material for: Saussurea involucrata CML6 Enhances Freezing Tolerance by Activating Antioxidant Defense and the CBF-COR Pathway in Plants
Source: Plants (Basel). 2025 Aug 1;14(15):2360. doi: 10.3390/plants14152360 (PMC12348903; doi:10.3390/plants14152360)
Supplement: Supplementary file 1 [file plants-14-02360-s001.zip › plants-3785355-supplementary.pdf]

Supplementary materials

Table S 1 Primers used in this study

Name	Primer	Purpose
SiCML1-F	TTGACAAAGACGGAGATGGG	qRT-PCR
SCML1-R	GCTCAGCTTCAGTAGGATTCTG	
SiCML2-F	AGGAGCTTGGAACAGTGATG	
SiCML2-R	TCCTTCATCTTCCTTGCCATC	
SiCML3-F	GATATTTGAGGTGGCGGATTTG	
SiCML3-R	ACGCTTATCTTCCCATCACC	
SiCML4-F	GCATCACTACAAAGGAGCTTG	
SiCML4-R	ATAGTACCGTTTCCATCAGCG	
SiCML5-F	TTCCCCTAAAACCCCATCAC	
SiCML5-R	TTGCCGTCCCTGTTCTTG	
SiCML6-F	CGACTCTTTCCACAATACCCAC	
SiCML6-R	TTCACCACCATCTCCTTCTTC	
SiCML7-F	ATGGTTGAAGGATTGACACTTG	
SiCML7-R	GTTCATCATTTCTTGGACCTCT	
GAPDH-F	TAGCAAGGATGCTCCCATGTTCGT	
GAPDH-R	AAAGGAGCAAGGCAGTTGGTTGTG	
SiCML6-F	GGTACCTTGTCGAAGATGGTTGAAGGA	Gene cloning
SiCML6-R	TCTAGATTGTTTACGAGGCACTCATCA	
qRT-AtCBF1-F	ACTTCGCTGACTCGGCTTGG	qRT-PCR
qRT-AtCBF1-R	ACGCACCTTCGCTCTGTTCC	
qRT-AtCBF2-F	GGAATCAACCTGTGCCAAGGAA	
qRT-AtCBF2-R	CCAACATCGCCTCTTCATCCATAT	
qRT-AtCBF3-F	TTCCTCAGGCGGTGATTATATTCC	
qRT-AtCBF3-R	CTCCGACGAACTCCTCTGTATATTG	
qRT-AtCOR15a-F	CCCGTCATCCAAGAACAACC	
qRT-AtCOR15a-R	CTGAGCTTTTCGGGGTTCCT	

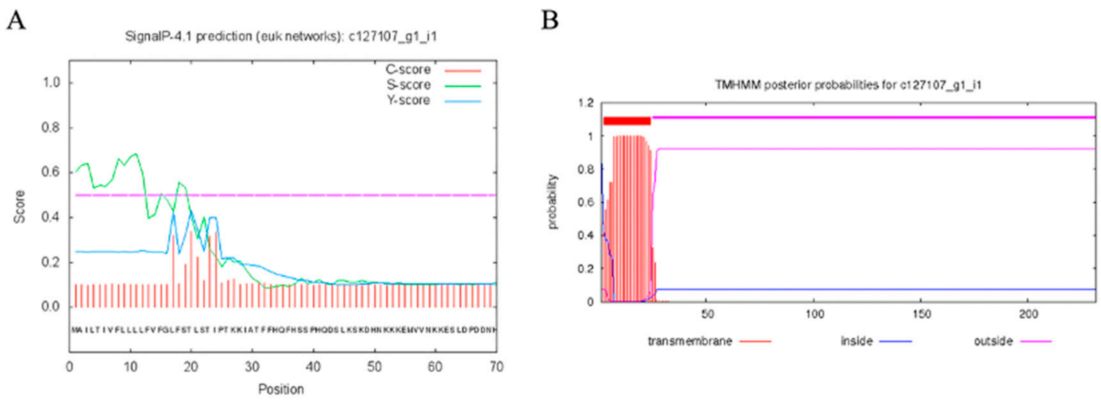

Figure S1. Signal peptide (A) and transmembrane domain analysis (B) of SiCML6

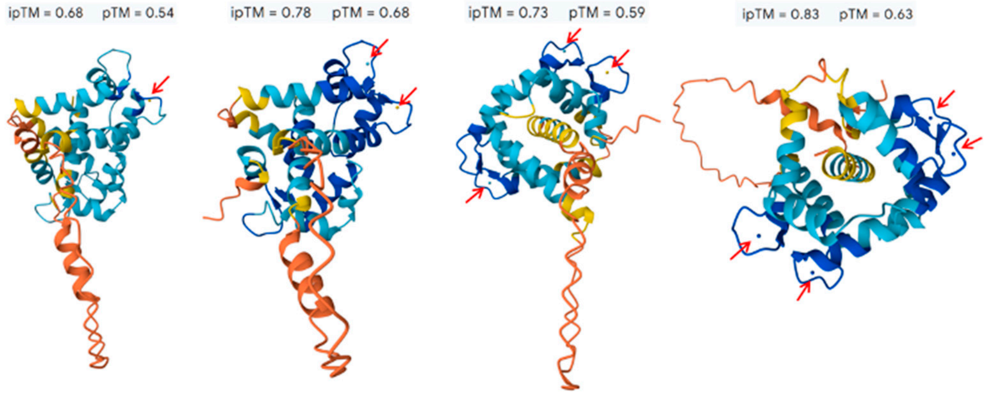

Figure S2. Molecular docking of SiCML6 with varying numbers of calcium ions

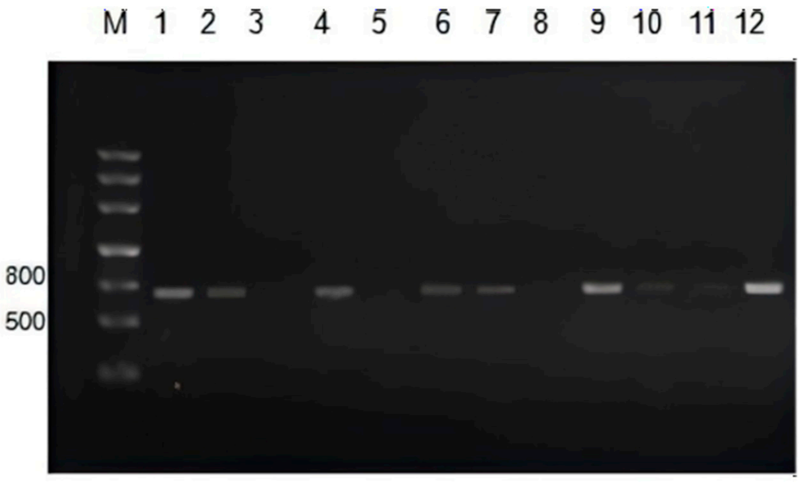

Figure S3. Characterization of transgenic Arabidopsis thaliana through DNA PCR. Numbers 1-22 indicate individual transgenic plant lines.

Sequence S1. SiCML6 CDS sequence.

ATGGCTATTTTAACCATCGTCTTTCTTCTTCTCCTCTTCGTTTTTGGCCTTTTTTCGACTC
TTTCCACAATACCCACAAAAAAAATCGCTACTTTTTTTCATCAATTTCACTCTTCACCC
CATCAAGATTCACTCAAAAGTAAGGATCACAACAAGAAGAAGGAGATGGTGGTGAA
CAAGAAAGAGAGTTTGGATCCTGATGATAATCATTCGAGGGAAAGAAAAGCAGAGT

TGAAGAGTGTTTTCGCCACTTTCGACAAAAACAAAGATGGGTTCATCACAAAACAAG
AGTTGAGTGATTCACTCAAGAACATCGGGATCTCAACCAGTGAAAAAGATGTGATTG
AAATGGTACAAAAAGTTGATGTGAATGGAGATGGGTTGATTGATTTCGATGAGTTTTG
CGAGCTTTTCGAGTCGATGATGAGGCGAGAAGACCATAATAAGATCGAGAATCTTGA
TCATGAAGATGGGGATTTGAAGGATGCTTTTGATGTATTTGATGGGGATAAAAATGG
GCTCATAAGTGTTGAGGAATTGGGGTTGGTTTTGGATTCTTTGGGGTTTAAAGAAGGG
AAAACGTTGGAGGATTGCAAGATGATGATAAGCAAAGTTGATATTGATGGTGATGGT
ATGATCAATTTTCATGAGTTCAAGAACATGATGAAGAATGGTGTTGGTCTTGTTTCGG
TTTCTTGA

Sequence S2. SiCML6 Protein sequence.
MAILTIVFLLLLFVFGLFSTLSTIPTKKIATFFHQFHSSPHQDSLKSKDHNKKKEMVVNKKES
LDPDDNHSRERKAELKSVFATFDKNKDGFITKQELSDSLKNIGISTSEKDVIEMVQKVDVN
GDGLIDFDEFCELFESMMRREDHNKIENLDHEDGDLKDAFDVFDGDKNGLISVEELGLVL
DSLGFKEGKTLEDCKMMISKVDIDGDGMINFHEFKNMMKNGVGLVSVS-
